# Impact of natural light exposure on delirium burden in adult patients receiving invasive mechanical ventilation in the ICU: a prospective study

**DOI:** 10.1186/s13613-019-0592-x

**Published:** 2019-10-17

**Authors:** Roland Smonig, Eric Magalhaes, Lila Bouadma, Olivier Andremont, Etienne de Montmollin, Fatiah Essardy, Bruno Mourvillier, Jordane Lebut, Claire Dupuis, Mathilde Neuville, Mathilde Lermuzeaux, Jean-François Timsit, Romain Sonneville

**Affiliations:** 1 Department of Intensive Care Medicine and Infectious Diseases, Bichat-Claude Bernard University Hospital, Assistance Publique - Hôpitaux de Paris, 46 Rue Henri Huchard, 75018 Paris, France; 2 UMR 1137, IAME Team 5, DeSCID: Decision SCiences in Infectious Diseases, Control, and Care, INSERM/Université Paris Diderot, Sorbonne Paris Cité, Paris, France; 3 Université de Paris, UMR 1148, Laboratory for Vascular and Translational Science, Paris, France

**Keywords:** Delirium, Light, Agitation, Hallucinations, Antipsychotics

## Abstract

**Objective:**

To determine whether potential exposure to natural light via windows is associated with reduced delirium burden in critically ill patients admitted to the ICU in a single room.

**Design:**

Prospective single-center study.

**Setting:**

Medical ICU of a university hospital, Paris, France.

**Patients:**

Adult patients receiving invasive mechanical ventilation.

**Methods:**

Consecutive patients admitted to a single room with (LIGHT group) or without (DARK group) exposure to natural light via windows were evaluated for delirium. The primary endpoint was the incidence of delirium. Main secondary endpoints included incidence of severe agitation intervened with antipsychotics and incidence of hallucinations.

**Results:**

A total of 195 patients were included (LIGHT group: *n* = 110; DARK group: *n* = 85). The incidence of delirium was similar in the LIGHT group and the DARK group (64% vs. 71%; relative risk (RR) 0.89, 95% CI 0.73–1.09). Compared with the DARK group, patients from the LIGHT group were less likely to be intervened with antipsychotics for agitation episodes (13% vs. 25%; RR 0.52, 95% CI 0.27–0.98) and had less frequent hallucinations (11% vs. 22%; RR 0.49, 95% CI 0.24–0.98). In multivariate logistic regression analysis, natural light exposure was independently associated with a reduced risk of agitation episodes intervened with antipsychotics (adjusted odds ratio = 0.39; 95% CI 0.17–0.88).

**Conclusion:**

Admission to a single room with potential exposure to natural light via windows was not associated with reduced delirium burden, as compared to admission to a single room without windows. However, natural light exposure was associated with a reduced risk of agitation episodes and hallucinations.

## Background

Delirium is a common complication in the ICU, occurring in up to 80% of invasively mechanically ventilated patients. It is characterized by a disturbance of attention with a change in cognition and a fluctuating course, with or without associated hyperactive symptoms (i.e., agitation and hallucinations) [1], the hypoactive phenotype being much more prevalent than the hyperactive one in recent studies conducted in ICU patients [2]. Delirium in the ICU is associated with adverse outcomes, higher ICU and hospital length of stay and costs, and a higher risk of cognitive impairment in survivors [3]. Risk factors include mainly non-modifiable factors, i.e., greater age and dementia, prior coma, pre-ICU emergency surgery or trauma, and severity of illness. Potentially modifiable factors for delirium are scarce and include non-environmental factors, i.e., benzodiazepine exposure and blood transfusion [1].

The use of the multicomponent ABCDEF bundle (i.e., the assessment, prevention, and management of pain, spontaneous awakening and breathing trials, choice of analgesia and sedation, delirium assessment, early mobility and exercise, and family engagement and empowerment) was shown to be associated with significant and clinically meaningful improvements in outcomes including survival, mechanical ventilation use, coma, delirium, restraint-free care, ICU readmissions, and post-ICU discharge disposition [4, 5].

It remains unclear whether intensive care environment affects the course of delirium and outcomes. Loss of exposure to natural light is associated with circadian rhythm disruption that may impact delirium burden and outcomes in the critically ill [6, 7]. Other mechanisms such as reduced views of natural surroundings or direct alerting effects of light may also play a role. Recent studies conducted in patients with or without acute brain injury suggest no clear association between natural light exposure and mortality, functional outcome or costs of in-hospital care [8, 9]. Moreover, data on the relation between light exposure and delirium in the ICU are scarce. A single-center case–control study suggested a reduction of delirium incidence and duration in patients admitted to single rooms with windows, as compared to historical controls admitted to a general ICU with wards [10]. Bright-light therapy in the daytime was also tested to reduce the incidence of delirium in the ICU. In a multicenter randomized control trial, high-intensity dynamic light application, as compared with normal lighting, did not reduce the cumulative incidence of delirium [11].

The aim of the present study was to investigate the impact of potential natural light exposure via windows on delirium burden in mechanically ventilated patients admitted to the ICU in a single room.

## Methods

### Study design

This prospective, observational, single-center study was conducted in the 26-bed medical intensive care unit of the Bichat-Claude Bernard university hospital, *Assistance Publique Hôpitaux de Paris*, Paris, France, between January 3rd, 2016 and January 3rd, 2017. The local ethical committee CEERB Paris Nord (IRB 0000647, study number 16-026) approved the study. We included consecutive patients receiving invasive mechanical ventilation (MV) for an expected duration of at least 2 days. We excluded patients with a MV duration less than 2 days, patients who had been ventilated for more than 1 day in another unit before admission to our ICU, acute brain injury patients and patients with preexisting conditions known to interfere with delirium assessment (e.g., blindness, deafness, and overt dementia).

### Intensive care unit

The medical ICU of the Bichat-Claude Bernard university hospital is a 26-bed unit, composed of two acute care units (10 beds each) and one medium care unit (6 beds). Each acute care unit is composed of four rooms without natural light exposure (DARK rooms) and six rooms with natural light exposure via windows (LIGHT ROOMS). A detailed map of the three units of the ICU is provided in Additional file 1: Online resource 1. As there is no particular policy of admission with regards to exposure to natural light, patients admitted to one of the acute care units are usually assigned to the “first available room”. Pictures of DARK rooms without natural light exposure and LIGHT rooms with natural light exposure via windows are provided in Additional file 1: Online resource 2.

### Management

Patients under invasive mechanical ventilation were managed according to a written protocol (Additional file 1: Online resource 3), including use of common sedative (i.e., midazolam, propofol, dexmedetomidine) and analgesic (i.e., fentanyl or sufentanil) drugs, monitoring of sedation and delirium with Richmond Agitation–Sedation Scale (RASS) and the Intensive Care Delirium Screening Checklist (ICDSC), respectively [12, 13]. Daily sedation stops were performed at 10:00 a.m., with spontaneous awakening trials and spontaneous breathing trials, as previously described by others [14, 15]. Antipsychotics (i.e., haloperidol) were only administered to non-cooperative patients developing agitation after discontinuation of sedation, defined by a positive RASS and frequent non-purposeful movements and/or attempts to remove tubes and catheters [1, 16]. Haloperidol (diluted in 0.9% saline) was administered intravenously at an initial dose of 1–4 mg until reduction of agitation, with repeated doses every 4–6 h, as needed, up to a maximum dose of 20 mg per day.

### Delirium assessment

All nurses received theoretical training on ICDSC under supervision of the medical team. Routine screening for delirium was implemented 6 months before study initiation. The ICDSC was applied to each patient twice a day, 7 days a week, by the nurse in charge of the patient.

Patients were categorized as “comatose” on a given day if they could not be assessed for delirium because of a low RASS (i.e., ≤ − 4) during the whole day. Any day with at least one score on the ICDSC ≥ 4 was considered to be a day of delirium. Delirium occurrence was defined as the presence of delirium for at least 2 consecutive days during ICU stay. Any day with a positive RASS and a pharmacologic intervention with antipsychotics to treat hyperactive symptoms was considered to be a day of agitation. Hallucination occurrence was defined by any day with at least one episode of hallucinations, as scored on the ICDSC.

### Outcomes

The primary outcome was the cumulative incidence of delirium, defined as the presence of delirium for at least 2 consecutive days during ICU stay. Secondary outcomes were the duration of delirium, duration of coma, use of antipsychotics to treat agitation, the incidence of hallucinations, the incidence of self-extubation, duration of mechanical ventilation (MV), ICU and hospital length of stay, ICU and hospital mortality.

### Collected data

Data were collected prospectively, on a daily basis. ICU admission characteristics included age, gender, the Charlson comorbidity index [17], the Simplified acute physiology score II (SAPS2) [18], and the sequential organ failure (SOFA) score [19]. Other collected variables were selected based on their expected association with delirium and short-term outcome. These variables included history of dementia, history of alcohol abuse, chronic obstructive pulmonary disease (COPD), type of admission (medical vs. surgical), and sepsis as the admission diagnosis. Data on the use of sedative and hypnotics agents (molecule(s) and dose) and antipsychotic prescription (i.e., haloperidol) to treat agitation during ICU stay were also collected. RASS scores and occurrence of delirium were noted every day until death, ICU discharge or day 14. Measurements of daylight exposure according to patients’ view were performed post hoc, using the lightmeter^®^ 2.0 (Elena Polyanskaya©) smartphone application.

### Statistical analysis

Data are presented as median (interquartile range) or numbers (percentage) for continuous and categorical variables, respectively. Patients were divided into two groups, depending on whether ICU admission was done in a single room with (LIGHT group) or without (DARK group) natural light exposure via windows. Continuous variables were compared between groups using the Student’s *t*-test for continuous, normally distributed data and the Mann–Whitney *U* test for continuous, skewed data. The Chi-square test was used to compare categorical data.

Sample size calculation was based on published delirium rates for intubated in medical ICU patients [20]. To achieve a power of power 80% to detect a decrease of delirium from 80 to 60% (two-sided test, alpha = 0.05) and considering that LIGHT to DARK room ratio is 3/2 in our ICU, we calculated that a total of 180 patients would be necessary. The association between daylight exposure and delirium was explored using multivariate logistic regression analysis. Clinically relevant factors and other factors associated with delirium in univariate analysis (*p* < 0.2) were entered in a multivariate model. Two-by-two interactions and collinearity between variables were tested. All statistical analyses were performed using Statview. A two-sided *p*-value less than 0.05 was deemed significant. Comparisons of daylight exposure between LIGHT and DARK rooms were performed post hoc by repeated measures ANOVA.

## Results

Between January 3rd, 2016 and January 3rd, 2017, a total of 854 patients were admitted to one of the acute care units of our ICU, of whom 440 received invasive mechanical ventilation, including 352 for more than 48 h. After exclusion of 157 patients, 195 patients were included (DARK group *n* = 95, LIGHT group *n* = 110). A flowchart is detailed in Additional file 1: Online resource 4. Baseline characteristics are presented in Table 1. Patients were predominantly males [age 60 (50–69) years] with SAPS 2 and SOFA scores of 51 (36–64) and 9 (7–11), respectively. Main reasons for ICU admission were acute respiratory failure or sepsis. Baseline characteristics were comparable between the DARK and the LIGHT groups, with the exception of the proportion of medical admissions that tended to be higher in the LIGHT group than in the DARK group.

**Table 1:** Baseline characteristics

Variable	All (*n* = 195)	Dark (*n* = 85)	Light (*n* = 110)	*p*
Demographics				
Age, years	60 (50–69)	61 (51–68)	60 (48–70)	0.85
Male gender	135 (69)	58 (68)	77 (70)	0.79
History				
Immunodepression	25 (13)	12 (14)	13 (12)	0.64
Chronic antipsychotic use	13 (7)	6 (7)	7 (6)	0.84
Hypertension	93 (48)	38 (45)	55 (50)	0.46
Current smoking	68 (35)	31 (36)	37 (34)	0.68
COPD	37 (19)	20 (24)	17 (15)	0.15
Drug abuse	10 (5)	5 (6)	5 (5)	0.67
Benzodiazepine use	31 (16)	12 (14)	19 (17)	0.55
Cirrhosis	14 (7)	5 (6)	9 (8)	0.54
Alcohol abuse	46 (24)	24 (28)	22 (20)	0.18
Diabetes	45 (23)	18 (21)	27 (25)	0.58
Mild cognitive impairment	2 (1)	0 (0)	2 (2)	0.21
Charlson comorbidity index	2 (1–4)	2 (1–4)	2 (1–4)	0.20
Medical admission	134 (69)	52 (61)	82 (75)	0.05
Sepsis at admission	124 (64)	54 (64)	70 (64)	0.99
SOFA score	9 (7–11)	8 (6–11)	9 (7–11)	0.22
SAPS 2	51 (36–64)	52 (36–61)	50 (36–65)	0.64
ICU admission diagnosis				
Respiratory failure or sepsis	105 (54)	46 (54)	59 (54)	0.85
Cardiogenic shock	32 (16)	13 (15)	19 (17)	0.71
Cardiothoracic surgery	26 (13)	13 (15)	13 (12)	0.48
Other	32 (16)	13 (15)	19 (17)	0.71

Data are median (interquartile range) or numbers (percentage)

*COPD* chronic obstructive pulmonary disease, *SOFA* Sepsis Organ Failure Assessment, *SAPS 2* Simplified Acute Physiology Score 2

Opioids and hypnotics exposure mainly consisted of fentanyl and midazolam, in accordance with the local sedation protocol. There was no difference between groups in terms of opioids and hypnotics exposure, as shown in Table 2. Cumulative doses of fentanyl, midazolam, and propofol tended to be lower in the LIGHT group, as compared to the DARK group. Overall, 33 (17%) patients from the DARK group were secondarily transferred to another room with windows.

**Table 2:** Use of opioids and hypnotics during ICU stay

Variable	All (*n* = 195)	Dark (*n* = 85)	Light (*n* = 110)	*p*
Opioids				
Morphine				
Patients	37 (19)	16 (19)	21 (19)	0.96
Cumulative dose, mg	528 (294–948)	384 (276–792)	696 (276–1176)	0.61
Fentanyl				
Patients	159 (82)	68 (80)	91 (83)	0.62
Cumulative dose, mg	7.2 (3.6–14.4)	9.6 (4.8–16.2)	6 (3.6–13.2)	0.09
Hypnotics				
Midazolam				
Patients	167 (86)	71 (84)	96 (87)	0.46
Cumulative dose, mg	240 (120–480)	276 (120–480)	216 (120–468)	0.19
Propofol				
Patients	76 (39)	32 (38)	44 (40)	0.74
Cumulative dose, mg	4560 (2160–7200)	5760 (2400–8400)	3240 (1800–6000)	0.11
Dexmedetomidine				
Patients	29 (15)	13 (15)	16 (15)	0.88
Cumulative dose, mg	2.6 (1.2–3.7)	2.6 (2.3–2.8)	3.1 (1.1–4.3)	0.78

Data are median (interquartile range) or numbers (percentage)

*mg* milligram

Main outcomes are presented in Table 3. The cumulative incidence of delirium in the whole cohort was 67% (120/179 patients) and duration of delirium was 3 (1–7) days. A total of 32 (18%) patients were intervened with antipsychotics for agitation during ICU stay. Those patients had higher maximum RASS scores during ICU stay than patients who did not receive antipsychotics [2 (2–3) vs. 0 (0–2), *p* < 0.01]. The cumulative incidence of delirium was not different between the LIGHT group and the DARK group (64% vs. 71%; relative risk (RR) 0.89, 95% confidence interval (95% CI) 0.73–1.09). Compared with the DARK group, patients from the LIGHT group were less likely to be intervened with antipsychotics for agitation episodes (13% vs. 25%; RR 0.52, 95% CI 0.27–0.98) and had less frequent episodes of hallucinations (11% vs. 22%; RR 0.49, 95% CI 0.24–0.98). Other secondary outcomes, including self-extubation, ICU and hospital mortality rated did not differ between groups. Data on RASS scores during ICU stay are presented in Additional file 1: Online resource 5.

**Table 3:** Main outcomes

Variable	All (*n* = 195)	Dark (*n* = 85)	Light (*n* = 110)	*p*
Primary outcome				
Delirium cumulative incidence^a^	120/179 (67)	55/77 (71)	65/102 (64)	0.28
Secondary outcomes				
Duration of delirium, days^a^	3 (1–7)	3 (1–7)	3 (1–6)	0.43
Duration of coma, days	2 (1–5)	2 (1–5)	2 (1–5)	0.76
Patients intervened with antipsychotics to treat agitation^a^	32/179 (18)	19/77 (25)	13/102 (13)	0.04
Hallucinations^a^	28/179 (16)	17/77 (22)	11/102 (11)	0.04
Self-extubation	15 (8)	5 (6)	10 (9)	0.40
Duration of ventilation, days	7 (3–13)	7 (3–13)	7 (3–12)	0.89
ICU LOS, days	12 (7–19)	12 (8–18)	11 (7–20)	0.67
Hospital LOS, days^b^	25 (14–49)	26 (15–53)	24 (14–43)	0.41
ICU mortality	47 (24)	20 (24)	27 (25)	0.87
Hospital mortality^b^	60/182 (33)	24/81 (30)	36/101 (36)	0.74

Data are median (interquartile range) or numbers (percentage)

^a^16 patients with coma during the whole ICU stay were excluded from analysis

^b^13 patients transferred to other hospitals during ICU stay were excluded from analysis

*ICU* intensive care unit, *LOS* length of stay

Sensitivity analysis performed after exclusion of patients initially admitted to a room without windows and secondarily transferred to a room with windows (exclusion of 32 patients at risk) revealed no change in the overall risk of delirium [38/58 (66%) patients in the DARK group versus 54/89 (61%) in the light group; RR 0.93, 95% CI 0.72–1.19].

In multivariate logistic regression analysis (Table 4), natural light exposure via windows was independently associated with a reduced risk of agitation episodes intervened with antipsychotics (adjusted odds ratio = 0.39; 95% CI 0.17–0.98).

**Table 4:** Factors associated with the use of antipsychotics to treat agitation, multivariate analysis

Variable	Adjusted odds ratio^a^	95% confidence interval
Admission to a room with window (LIGHT group)	0.39	0.17–0.88
Medical admission	4.55	1.43–14.26

^a^Variables associated in univariate analysis and tested in the multivariate model: COPD, alcohol abuse, medical admission, admission to a room with window

**Fig. 1:**
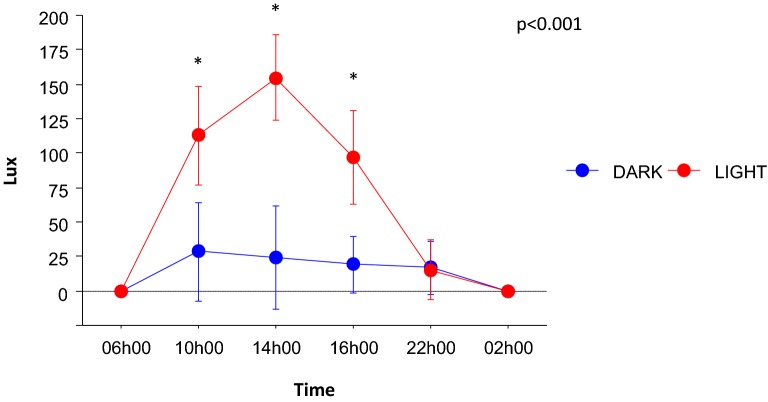
Illuminance (lux) measured at different hours of the day in rooms with windows (LIGHT group) or without windows (DARK group). Data presented as mean and 95% confidence interval. *p*-value was obtained by repeated measures ANOVA for overall significance of the difference in time course of lux (time * group interaction). *p < 0.05 for LIGHT versus DARK group

A post hoc analysis demonstrated a significant difference of illuminance between DARK rooms and light rooms. There was a significant interaction between the room effect and the hour of measurement effect, suggesting that LIGHT rooms were associated with preserved circadian variations of natural light (Fig. 1).

## Discussion

In adult mechanically ventilated patients in single rooms, potential natural light exposure via windows did not change delirium incidence, as compared to admission in a room without windows. However, admission to a room with windows seemed to have a protective effect on severe agitation episodes intervened with neuroleptics and hallucinations.

Data on the impact of natural light exposure during ICU stay on outcomes are scarce, mainly derived from single-center retrospective studies [8, 9, 21]. Taken together, these studies suggest no association between natural light exposure and mortality, functional outcome or costs of in-hospital care, in both brain-injured and non-brain injured patients. To date, only one single-center study suggested a potential benefit on the effect of natural light exposure and delirium in ICU [10]. Although this study was the first to suggest that ICU environment may influence the course of delirium, it also had limitations, including a small population, a rather low delirium rate reflecting a low-severity population, and a before–after design. ICU delirium was compared between an ICU with wards and a single-room ICU with, among others, improved daylight exposure. Therefore, the exact impact of daylight exposure on delirium in this study appears difficult to address. Compared to previously published data, our large study had significant strengths to accurately assess the exact impact of potential exposure to natural light via windows on ICU delirium. We observed a high delirium burden, in accordance with previous multicenter studies [20], we only included severe patients requiring at least 2 days of invasive ventilation and all patients benefited from a single room during ICU stay.

Previous studies suggested an impact of light before ICU admission on the outcome, with shorter photoperiods before admission in the ICU being associated with better outcomes [21]. However, a retrospective analysis of a multicenter study found no association between preadmission sunlight exposure and delirium incidence in ICU patients [22]. In our study, because we included consecutive patients under mechanical ventilation over a 1-year period, we believe that the risk of bias due to pre-admission sunlight exposure was low.

Dynamic light exposure has also been suggested to impact delirium and outcomes. A large multicenter randomized trial on the influence of high-intensity dynamic light application on delirium in ICU patients was terminated prematurely for futility after an interim analysis [11]. In this study, almost all included patients benefited from an ICU room with windows, allowing exposure to natural light. Second, antipsychotics were prescribed in a large proportion of patients, suggesting that their use was largely prophylactic, and not limited to treat severe episodes of agitation. Therefore, as acknowledged by authors, the impact of bright-light therapy on outcome should be assessed as part of a multicomponent strategy, rather than as a single intervention.

In our study, delirium incidence was high, and it is possible that the potential beneficial effects of natural light exposure were counterbalanced by the severity of illness. Antipsychotics were administered only in case of severe agitation, in accordance with the current guidelines [1]. Of note, the rate of patients intervened with antipsychotics was similar to that observed in a recent observational study on antipsychotic use in ICU patients with delirium [16].

One interesting observation from our study is that a stay in a room with potential exposure to natural light is associated with a reduction in the incidence of severe agitation episodes intervened with antipsychotics, as compared to admission to a room without windows. This association, which remained significant after adjusting for confounders, suggests a beneficial role of natural light exposure to prevent or treat hyperactive delirium. To our knowledge, this is the first report of such an independent association in the ICU setting, and this should be further investigated and validated. Hypotheses as to why preservation of natural light exposure would reduce agitation include reduced circadian rhythm disruption, and preserved space and time orientation. Moreover, other pathways modulating melatonin secretion may also be involved.

Our study has several strengths, including a large number of consecutive patients under invasive mechanical ventilation, with high severity scores and a high delirium incidence during ICU stay. Patients were all managed according to a strict sedation protocol, including daily sedation stops and delirium assessment with a validated tool. The distribution of rooms with or without windows in the ICU allowed avoiding other sources of environmental bias, such as noise. The choice of 2 consecutive days of positive ICDSC to define delirium was decided to minimize the possible effect of residual sedation, as rapidly reversible sedation-related delirium may not carry the same poor prognosis as persistent delirium [23].

Our study also has limitations. The single-center design limits the external validity of our findings. No validity assessment of delirium status was performed to detect potential inter-observer variability associated with use of the ICDSC scale. There was no randomization and admissions were made daily “in the first available bed”, therefore causality remains to be demonstrated. Moreover, assignments to rooms were done by persons aware of the study, which may obviously introduce bias. We observed a slight imbalance of admission characteristics between the two groups, with a higher proportion of surgical patients admitted to a room without windows. As compared to rooms without windows, rooms with windows are likely to have a higher natural light intensity and exposure duration. Although continuous light intensity and exposure duration measurements were not performed in this study, serial measurements performed at different time of day revealed higher light exposure in rooms with windows, as compared to rooms without windows. At last, some patients admitted to a room without windows were transferred to a room with a window during their ICU stay. Because too few patients received this intervention, it is difficult to state to what extent this may have impacted delirium burden.

## Conclusion

Admission to a single room with potential exposure to natural light via windows was not associated with reduced delirium burden, as compared to admission to a single room without windows. However, natural light exposure was associated with a reduced risk of agitation episodes and hallucinations. These findings deserve validation and should be considered exploratory.

## Supplementary information

**Additional file 1: Online resource 1.** Description of the ICU. **Online resource 2.** Pictures of DARK and LIGHT rooms. **Online resource 3.** Protocol for sedation and weaning from mechanical ventilation (french version). **Online resource 4.** Study flowchart. **Online resource 5.** RASS scores during ICU stay.

## Data Availability

The datasets used and/or analyzed during the current study are available from the corresponding author on reasonable request.
