# Supplementary material for: Impact of natural light exposure on delirium burden in adult patients receiving invasive mechanical ventilation in the ICU: a prospective study
Source: Ann Intensive Care. 2019 Oct 17;9:120. doi: 10.1186/s13613-019-0592-x (PMC6797676; doi:10.1186/s13613-019-0592-x)
Supplement: Supplementary file 1 — Additional file 1: Online resource 1. Description of the ICU. Online resource 2. Pictures of DARK and LIGHT rooms. Online resource 3. Protocol for sedation and weaning from mechanical ventilation (french version). Online resource 4. Study flowchart. Online resource 5. RASS scores during ICU stay. [file 13613_2019_592_MOESM1_ESM.docx]

**Impact of natural light exposure on delirium burden in adult patients receiving invasive mechanical ventilation in the ICU: a prospective study**

^1^Roland Smonig (M.D.), ^1^Eric Magalhaes (M.D.), ^1,2^Lila Bouadma (M.D., Ph.D.), ^1^Olivier Andremont (M.D.), ^1,2^Etienne de Montmollin (M.D.), ^1^Fatiah Essardy (RN), ^1^Bruno Mourvillier (M.D.), ^1^Jordane Lebut (M.D.), ^1^Claire Dupuis (M.D., Ph.D.), ^1^Mathilde Neuville (M.D.), ^1^Mathilde Lermuzeaux (M.D.), ^1,2^Jean-François Timsit (M.D., Ph.D.), ^1,3^Romain Sonneville (M.D., Ph.D.)

**ELECTRONIC SUPPLEMENTARY MATERIAL (ESM)**

Online resource 1. Description of the ICU

**Online resource 2. Pictures of DARK and LIGHT rooms**

**Online resource 3. Protocol for sedation and weaning from mechanical ventilation (french version)**

**Online resource 4. Study flowchart.**

**Online resource 5. RASS scores during ICU stay.**

|  | **All**  **N=195** | **Dark**  **N=85** | **Light**  **N=110** | **p-value** |
| --- | --- | --- | --- | --- |
| RASS, median (IQR) | 1 [0; 2] | 0 [0; 2] | 1 [0; 2] | 0.13 |
| RASS > 0 | 104 (53) | 40 (47) | 64 (58) | 0.12 |
| Intervened with antipsychotics | 29 (15) | 17 (20) | 12 (11) | 0.08 |
| Not intervened with antipsychotics | 75 (38) | 23 (27) | 52 (47) | 0.004 |
| RASS > 1 | 77 (39) | 29 (34) | 48 (44) | 0.18 |
| Intervened with antipsychotics | 25 (13) | 14 (16) | 11 (10) | 0.18 |
| Not intervened with antipsychotics | 52 (27) | 15 (18) | 37 (34) | 0.01 |
